# The Diverse Genomic Landscape of Diamond–Blackfan Anemia: Two Novel Variants and a Mini-Review

**DOI:** 10.3390/children10111812

**Published:** 2023-11-15

**Authors:** Iordanis Pelagiadis, Ioannis Kyriakidis, Nikolaos Katzilakis, Chrysoula Kosmeri, Danai Veltra, Christalena Sofocleous, Stavros Glentis, Antonis Kattamis, Alexandros Makis, Eftichia Stiakaki

**Affiliations:** 1 Department of Pediatric Hematology-Oncology, University Hospital of Heraklion, School of Medicine, University of Crete, 71003 Heraklion, Greece; ipelagiadis@pagni.gr (I.P.); kyriakidis@med.uoc.gr (I.K.); katzilaher@yahoo.gr (N.K.); 2 Department of Pediatrics, University Hospital of Ioannina, Faculty of Medicine, School of Health Sciences, University of Ioannina, 45110 Ioannina, Greece; chrisa.kosmeri@gmail.com (C.K.); amakis@uoi.gr (A.M.); 3 Laboratory of Medical Genetics, “Aghia Sophia” Children’s Hospital, Medical School, National and Kapodistrian University of Athens, 11527 Athens, Greece; dveltra@med.uoa.gr (D.V.); csofokl@med.uoa.gr (C.S.); 4 Division of Pediatric Hematology-Oncology, First Department of Pediatrics, “Aghia Sofia” Children’s Hospital, Medical School, National and Kapodistrian University of Athens, 11527 Athens, Greece; sglentis@med.uoa.gr (S.G.); ankatt@med.uoa.gr (A.K.)

**Keywords:** Diamond–Blackfan anemia, splicing, splice variants, mutation, ribosome, ribosomal proteins, congenital bone marrow failure syndrome, phenotype, congenital abnormalities, GATA1 1 transcription factor

## Abstract

Diamond–Blackfan anemia (DBA) is a ribosomopathy characterized by bone marrow erythroid hypoplasia, which typically presents with severe anemia within the first months of life. DBA is typically attributed to a heterozygous mutation in a ribosomal protein (RP) gene along with a defect in the ribosomal RNA (rRNA) maturation or levels. Besides classic DBA, DBA-like disease has been described with variations in 16 genes (primarily in *GATA1*, followed by *ADA2* alias *CECR1*, *HEATR3*, and *TSR2*). To date, more than a thousand variants have been reported in RP genes. Splice variants represent 6% of identifiable genetic defects in DBA, while their prevalence is 14.3% when focusing on pathogenic and likely pathogenic (P/LP) variants, thus highlighting the impact of such alterations in RP translation and, subsequently, in ribosome levels. We hereby present two cases with novel pathogenic splice variants in *RPS17* and *RPS26*. Associations of DBA-related variants with specific phenotypic features and malignancies and the molecular consequences of pathogenic variations for each DBA-related gene are discussed. The determinants of the spontaneous remission, cancer development, variable expression of the same variants between families, and selectivity of RP defects towards the erythroid lineage remain to be elucidated.

## 1. Introduction

Diamond–Blackfan anemia (DBA), an inherited bone marrow failure (IBMF) syndrome, is a ribosomopathy identified in humans. With an incidence of seven cases per million live births, DBA is characterized by congenital erythroid hypoplasia (erythroblastopenia) with severely reduced erythroid colony-forming units (CFU-E) [1]. It typically presents with severe anemia within the first 2–3 months after birth (95% of patients diagnosed are <2 years of age; 99% are <5 years of age) [2]. To date, Next-Generation Sequencing (NGS) approaches, including gene panels and Whole Exome Sequencing (WES), are considered the method of choice for the identification of pathogenic and likely pathogenic (P/LP) variants located in ribosomal protein (RP) genes [3] (Table 1). In 22% of patients with DBA features, no P/LP variants are detected (or no genetic diagnosis is achieved), while some patients with P/LP variants in RP genes display syndromic characteristics or a preleukemic state with no signs of anemia [1,2,3]. Erythrocyte adenosine deaminase (eADA) levels are usually elevated in DBA, in contrast to a DBA phenocopy where biallelic *CECR1* variants result in ADA2 deficiency [3,4]. According to a position paper from the European Hematology Association, “DBA-like” disorders present with congenital red cell aplasia and intact ribosomal function [3,5]. Table 2 describes DBA-like phenotypes, which involve erythroblastopenia and normocytic hyporegenerative anemia with normal eADA levels and no defects in rRNA maturation [2]. Of note, less than half of DBA patients present with cardiac defects as well as dysmorphic features, including craniofacial malformations and the pathognomonic triphalangeal thumb. DBA patients have a 4.8-fold higher relative risk of developing solid tumors (including osteogenic sarcoma in children), myelodysplastic syndrome (MDS), and acute myeloid leukemia compared to the general population, with a cumulative incidence of 20% by 46 years of age [5,6,7]. The role of *TP53* in the latter trait of DBA is under investigation. The pathogenesis of DBA involves defects in ribosomal RNA (rRNA) maturation that provoke nucleolar stress, leading to the stabilization of p53 and activation of its targets, resulting in erythroid-specific cell cycle arrest and apoptosis. Defective erythropoiesis in DBA may also occur due to reactive oxygen species production by toxic free heme generated by the GATA1/HSP70 and globin/heme imbalance [2,8]. DBA is inherited as an autosomal dominant trait with variable penetrance that prompts genetic counseling. The treatment of DBA involves corticosteroids after the first year of life. In contrast, non-responders and patients who develop significant toxicity to treatment, as well as infants, require regular red blood cell (RBC) transfusions with prompt screening and therapy for iron overload. Hematopoietic stem cell transplantation (HSCT) from matched siblings or unrelated donors is an alternative to chronic transfusions and achieves 91% overall survival in young children with DBA [2,5].

Although the clinical features of most DBA patients may be typical (macrocytic anemia, reticulocytopenia, and normal bone marrow cellularity with an absence of erythroid progenitors), diagnostic challenges include overlapping and atypical manifestations of BMF with variable penetrance. Two patients with novel variants in RP genes are herein reported and discussed in the context of genetic and phenotypic heterogeneity of DBA.

## 2. Case Series

Both of the patients were referred to Pediatric Hematology–Oncology Units in Greece, and their clinical description adheres to the CARE (CAse REports) guidelines (https://www.equator-network.org/reporting-guidelines/care/, accessed on 20 September 2023).

**Case #1**. A 3-month-old male was admitted for poor feeding and marked pallor. Prenatal history was unremarkable. The infant was born at 35 weeks of gestation with low birth weight (normal for gestational age) and no dysmorphic features. At birth, there were no signs of anemia [hemoglobin (Hb) 13.6 g/dL], respiratory stress, or feeding problems. He presented at three months with severe anemia (Hb 1.8 g/dL). The patient developed cardiogenic shock despite immediate blood transfusions and required intensive care treatment. The initial diagnostic evaluation showed a reticulocyte index of 3.4% and normal bilirubin levels, while a smear of peripheral blood disclosed the presence of all white blood cell lineages with normal counts and morphology, increased platelets (644,000/μL) with variable size, and decreased red blood cells (RBC 0.47 million cells/μL, MCV 119.1 fL, MCH 38.3 pg, RDW 18.5%, reticulocytes 765/μL). The bone marrow aspirate revealed a complete absence of the erythroid lineage with the presence of hemophagocytic islets. Since there were no signs of hemolysis or blood loss, and a negative screening for infectious agents, congenital anemia associated with BMF, such as DBA, was highly suspected. Targeted Sanger sequencing of *RPS19*, *RPL11*, and *RPL5* genes was negative, and subsequent WES analysis revealed a de novo heterozygous pathogenic variant in the *RPS26* gene (NM_001029.5:c.181+1del; splice donor). The infant was placed on regular transfusions. At the last follow-up, he was receiving transfusions every three months with a pre-transfusion hemoglobin cut-off of 8–9 g/dL.

**Case #2**. A 4-month-old female presented with worsening pallor for three consecutive weeks. Personal and family histories were non-contributory. Development was normal for her age, and no congenital anomalies were present. Complete blood count revealed severe anemia (Hb 5.7 g/dL, MCV 97.8 fL, MCH 32.6 pg, reticulocytes 7200/μL, white blood cells 10,070/μL, and platelets 725,000/μL), while further laboratory investigations were significant only for low B12 vitamin levels (126 pg/mL). RBC transfusion and B12 administration resulted in a temporary hemoglobin increase (8.2 g/dL). As anemia relapsed and reticulocytopenia did not improve, the patient underwent a bone marrow aspiration, which showed normal cellularity with diminished erythroid precursors. Based on the latter findings, DBA was highly suspected. NGS was performed for 160 genes using the Illumina MiSeq System and SureSelect (Agilent, Santa Clara, CA, USA) and revealed a de novo heterozygous variant (c.156-1G>A) in the *RPS17* gene. This G>A substitution is a novel splice variant related to DBA. The infant received monthly RBC transfusions to maintain Hb levels > 8–9 g/dL until 12 months. After that age, prednisolone was introduced with a satisfying hemoglobin response without further transfusions.

Haploinsufficiency in RP genes results in defective RP biosynthesis and maturation, initiating ribosomal stress and apoptosis of erythroid progenitor cells. The two de novo and novel heterozygous pathogenic splice variants comprising *RPS26*:c.181+1del and *RPS17*:c.156-1G>A were conclusive in both cases. Splice site variants are commonly considered as P/LP and predicted to lead to a null effect. Further functional studies via either RNA or protein analysis are required to delineate the underlying mechanisms [15]. Doherty et al., 2010 were able to show that *RPS26* variants (including splice alterations) affect the function of proteins involved in rRNA processing in DBA patients. In particular, siRNA-mediated knockout of *RPS26*, encoding a component of the 40S ribosomal subunit, in HeLa cells led to decreased levels of 18S rRNA, impaired production of the small subunit and processing of the pre-rRNA, and accumulation of 43S, 26S, and 18S-E pre-rRNAs, thus indicating defects in cleavages at both ends of the 18S rRNA [16]. *RPS26* mutations have been identified as independent risk factors for short stature in DBA patients, which in turn reflects the cumulative steroid doses administered [17]. DBA-related *RPS26* mutations are not likely to display physical malformations; however, multiple physical abnormalities were recorded in a patient with a frameshift variant in *RPL26*. As shown by Gazda et al. (2012), the truncated RPS26 was unlikely to associate with the nascent ribosomal subunits, halting the synthesis of the large ribosomal subunits and resulting in low levels of the free 60S subunit, formation of half-mers in the polysomes, and a decrease in the amount of the precursors to large subunit rRNA [18]. On the other hand, RPS17 lies on top of the small ribosomal subunit near one end of the eukaryotic initiation factor eIF-2 binding site and in close vicinity to three out of five RPs (RPS13, RPS16, and RPS19) that are involved in eIF-2 binding [19].

## 3. Discussion

### 3.1. The DBA Genetic Landscape

**Figure 1:**
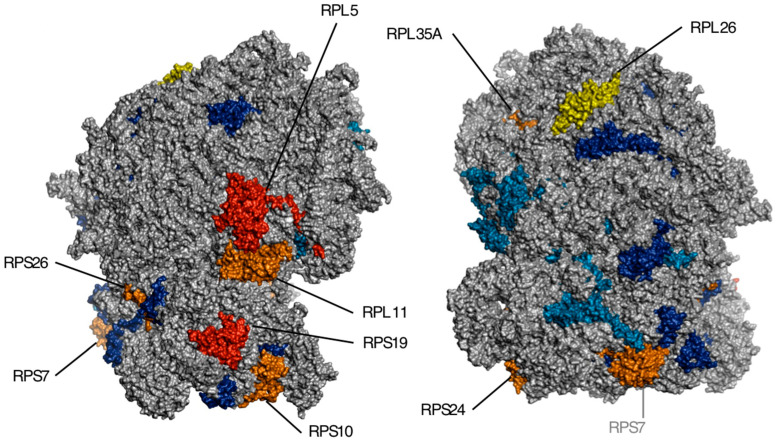
Known variations in RP genes associated with DBA.

**Figure 2:**
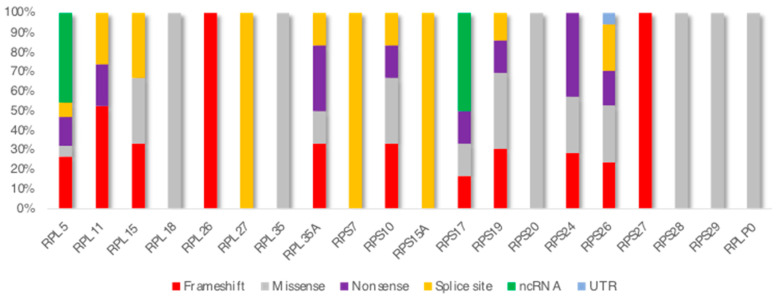
Molecular consequence of P/LP variations in RP genes.

To date, 1346 variations have been recorded in the ClinVar database (https://www.ncbi.nlm.nih.gov/clinvar/; accessed 18 October 2023), with P/LP variants accounting for 23% of records. According to ClinVar, the molecular consequences of variations in the DBA-related genes were missense mutations (48%), frameshift mutations (12%), splice variants (6%), and nonsense mutations (6%). SNVs are the most prevalent variation type (78.9%), followed by deletions (9.7%), insertions (5.9%), duplications (4.7%), and indels (0.9%). Variants typically reside in a single gene (99.4%), and the variation size is short (<50 bps; 98.9%). Among P/LP variants, frameshifts are common (31.1%), followed by missense (19.8%), non-coding RNA (ncRNA; 17.4%), nonsense (15.2%), splice site (14.3%), and untranslated region variations (UTR; 2.1%). Figure 1 illustrates all RPs in the literature with variations associated with DBA, and Figure 2 describes the molecular consequences of P/LP variations for each DBA-related RP gene. The results obtained from the ClinVar data investigation confirm the findings from previous DBA registry reports. *RPS19*, *RPL5*, *RPS26*, and *RPL11* are indisputably the most frequently mutated RP genes in all relevant publications [3,20,21]. EuroDBA reported that as many as 90% of the mutations occur in only six genes (*RPS19*, *RPL5*, *RPS26*, *RPL11*, *RPL35A*, and *RPS24*), which is in agreement with the findings of this survey (87%) [21]. At this point, we should clarify that the ClinVar and OMIM (Online Mendelian Inheritance in Man; https://www.omim.org/, accessed on 29 September 2023) approach was chosen due to the scarcity of published DBA registry reports on genetic defects, even though EuroDBA and DBAR have a satisfactory number of registered patients (1191 and 750, respectively) [21]. Obviously, OMIM does not provide a list of all the literature on the subject and ClinVar may not include all DBA-related variants ever diagnosed, and this is why we insist on growing DBA consortia and networks.

Designed with PyMOL and human 80S ribosome (Protein Data Bank structure 6IP5; https://www.rcsb.org/structure/6IP5, accessed on 17 October 2023) [22]. RPs in red: >50 P/LP variations; RPs in orange: 10–50 P/LP variations; RPL26 in yellow: 55 variations and 3 P/LP variations; RPs in dark blue: RPL15, RPL18, RPL27, RPL35, RPS15A, RPS17, RPS20, RPS27, RPS28, and RPS29: <10 P/LP variations in ClinVar; RPs in cyan: RPLP0 (uL10), RPL3 (uL3; chain 1E), RPL10 (uL16; 2D), RPL10A (uL1; 2I), RPL19 (eL19; 2L), RPL31 (eL31; 2X), RPL34 (eL34; 2a), and RPS11 (uS17; 2v)—mentioned in Ulirsch et al., 2018 [3]. (twenty-seven mutated RP genes in total including RPLP0).

### 3.2. DBA-like Disorders

Although DBA is mainly homogenous in phenotypic expression, 6% of cases that phenocopy DBA and DBA-like disorders harbor variants in non-ribosomal genes (such as *GATA1* and *ADA2*; Table 2) [3,23]. OMIM recognizes P/LP variants in *TSR2* and *HEATR3* genes as causative for DBA14 with MFD and DBA21, respectively [12,13]. TSR2 is involved in the processing and maturation of rRNA and binds to RPS26 (mutated in DBA10 correspondingly). Treacher Collins syndrome, which bears resemblance to the MFD accompanying DBA and displays rRNA reduction, is not characterized by increased risk for cancer, which could be partly linked to altered genetic functions, as with TSR2, which represses the transcription of NF-kappa B and seems to be involved in apoptosis [13,24]. Besides TSR2’s predominant role in producing adequate ribosome levels, it also seems necessary for erythroid lineage commitment through ribosome levels [25]. A significant amount of *GATA1* variations have been associated with DBA, indicating common pathways with RPs.

Haploinsufficiency in RPs results in an impaired translation of *GATA1* mRNA, thus mediating the erythroid defect in DBA. Cells bearing both RP and *GATA1* variants exhibit reduced proliferation and delayed erythroid differentiation. RP haploinsufficiency and the subsequent decrease in GATA1 levels affect the balance of globin-heme and result in the accumulation of free cytoplasmic heme in erythroid progenitors, increasing the p53-dependent apoptosis in these cells. Moreover, *GATA1* has 5′ UTR features that predict poor translation initiation rates, rendering its mRNA susceptible to ribosome deficiency [25,26]. *ADA2* is the only non-RP gene associated with DBA diagnosis with exome-wide significance, but it is unclear whether there are overlapping pathways [3]. Patients with *ADA2* variants present with a lack of erythroid precursors with maturation arrest in infancy, consistent with pure red cell aplasia, but they do not exhibit dysmorphic traits and, most importantly, they feature normal rRNA maturation [3,27,28]. However, if DBA is suspected, screening for *ADA2* variants should follow negative results for RP genes because HSCT seems to be effective in these patients [3]. NOP58 is required for the biogenesis of the 40S ribosomal subunit, and its defects are associated with growth arrest due to defects in early pre-rRNA processing events necessary for ribosome assembly, while specific *HEATR3* variants initially impair the nuclear import of RPL5 and eventually impair ribosome biogenesis [12,29]. DAAM1 is implicated in cell polarity mainly through the Wnt signaling pathway and is required for myocardial maturation and sarcomere assembly [30]. The EPO R150Q mutant displays only a mild reduction in affinity for its receptor. Still, its altered binding kinetics can lead to biased downstream signaling (reduced JAK2-mediated phosphorylation) and can thereby cause the ineffective stimulation of erythroid cell proliferation and differentiation. Unlike DBA, the latter entity can be effectively treated with EPO supplementation [3,31]. In the same context, the IKZF1 R381C variant was reported to have DBA-like features, an immune-related gastrointestinal phenotype, and an alteration in hematopoietic gene expression networks, while mutations in the *MPL* proto-oncogene of the thrombopoietin (TPO) receptor have been linked to elevated TPO and a DBA-like phenotype [14,32]. Loss-of-function variants in the *MYSM1* gene, which is a regulator of transcription, present with transfusion-dependent refractory anemia in early childhood in addition to mild thrombocytopenia and low NK- and B-cell counts. Notably, *MYSM1*-knockout mice manifest a BMF phenotype after oxidative DNA damage and increased p53 expression [33]. Similarly to *MYSM1*, *ZNF699* is involved in the regulation of transcription by RNA polymerase II by enabling DNA-binding transcription factors. Since 2021, it has been known to cause DEGCAGS syndrome, with anemia reported in 8 out of 14 diagnosed patients [34]. Loss-of-function variants in *NHEJ1*, which preferentially mediates the repair of DNA double-stranded breaks, are associated with dysmorphic facies and immunodeficiency, and roughly half of the patients demonstrate anemia–either autoimmune or following bone marrow aplasia [3,35]. Aside from sideroblastic anemia, variants in the *PUS1* gene, which plays an essential role in tRNA function and in stabilizing the secondary and tertiary structure of many RNAs, and in the *SLC25A38* gene, which is required during erythropoiesis and is essential for the biosynthesis of heme, were associated with a DBA-like phenotype. Notably, the knockout of both genes results in anemia [36,37]. Ultimately, de novo *TP53*-activating variants are well documented in inherited BMF syndrome 5, exhibiting in some cases DBA-like features [38]. Ultimately, the Table 2 data suggest the extension of investigations in non-RP genes in cases that resemble DBA.

### 3.3. The Correlation of Genetic Defects with Phenotype

The vast majority (96.8%) of DBA-associated P/LP variations correspond to ten RP genes, as illustrated in Figure 1. Although most DBA cases are sporadic, approximately 40–45% are familial, with the majority exhibiting autosomal dominant inheritance and incomplete penetrance [18]. In terms of inheritance, DBA14 with MFD is X-linked recessive, and DBA21 is autosomal recessive, but both entities account for only nine cases in the literature, i.e., less than 1% of reported cases [12,13]. Congenital hypoplastic normochromic and, usually, macrocytic anemia is routinely found in all DBA cases, while eADA is elevated in 8 out of 10 DBA-affected individuals [3,39]. Based on the ClinVar data (Table 1) and OMIM phenotypic series (105650; 606129; 610629; 612527; 612528; 612561; 612562; 612563; 613308; 613309; 614900; 615550; 615909; 300946; 606164; 617408; 617409; 618310; 618312; 618313; and 620072) for DBA, we estimated the prevalence of specific traits among DBA patients. Growth disorders are expected in one-third of the patients (mainly presenting as a failure to thrive, followed by short stature). Still, it is unknown how much anemia and corticosteroids affect this outcome. Intriguingly, a DBA cohort study reported short stature in 9% of patients before treatment initiation. The same study noted that nearly half of patients are expected to present with one or more congenital malformations at diagnosis [3]. Head, neck, and facial malformations are quite common among DBA patients. Mandibular deformities (primarily micrognathia followed by retrognathia) are expected in 17% of cases, clefts in 15–20%, and Cathie facies (short nose, broad nasal bridge, widely-spaced eyes, and thick upper lip) in approximately 10% of affected patients. Of interest, ear malformations seem to have been underestimated in DBA, with congenital aural atresia and microtia associated with 8.5% of the patients in OMIM. Triphalangeal thumbs are considered pathognomonic for DBA but occur only in roughly 15% of patients. Even though triphalangeal thumbs outnumber other skeletal malformations in DBA (accounting for 19% of the total), other disease entities should be ruled out [40]. Isolated thumb malformations are highly prevalent among patients with *RPL11* variants, while *RPL5* variants are associated with syndromic features, such as craniofacial, thumb, and heart deformities [41]. Patients with mutations in large ribosomal subunit protein (RPL) genes display significant correlations with the incidence of malformations, higher eADA levels, and more severe outcomes, compared to patients with mutations in small ribosomal subunit protein (RPS) genes [42]. A significant portion of DBA patients suffer from congenital heart conditions (15–20%), predominantly from either ventricular or atrial septal defect. Loss-of-function mutations in *RPS24* have been associated with congenital heart disease, even though they display incomplete penetrance [43]. Moreover, cardiac complications due to iron overload in transfusion-dependent children with DBA constitute a significant issue [44]. Regarding laboratory investigations, anemia can be accompanied by elevated HbF and reticulopenia, while neutropenia (though not profound) is under-recognized, accounting for 15% of reported DBA cases. Nine out of ten eligible DBA patients respond to glucocorticoid treatment, one-quarter of patients become steroid-dependent (especially patients with variants in *RPL5*, *RPL11*, and *RPS24* genes), and 11–25% of patients achieve remission spontaneously [3,45]. Risk for malignancies in DBA might be lower compared with other inherited BMF syndromes but malignancies affect a considerable portion of non-transplanted patients (5%). The observed/expected ratios of 4.8 for any malignancy, 44.7 for colon carcinoma, 9.4 for lung cancer, 42.4 for osteogenic sarcoma, 352 for MDS, and 28.8 for acute myeloid leukemia are high and impose challenges in genetic counseling [46]. DBA variants in patients with cancer/MDS refer mainly to *RPS19*, *RPL35A*, *RPL11*, *RPL5*, *RPS17*, and *GATA1* genes (18%, 13%, 10%, 5%, 3%, and 3%, respectively; 49% unknown) [47].

## 4. Conclusions

The individualized monitoring of DBA patients is crucial and requires an integrated multidisciplinary approach. Frequent complete blood counts and periodic bone marrow biopsy or aspiration are fundamental in the context of the early diagnosis of new cytopenias or BMF. Steroid-dependent and transfusion-dependent patients should be closely monitored for adverse effects and growth, while cancer surveillance imposes follow-up visits every 4–6 months for all DBA patients [48]. Genetic counseling is also essential and, hopefully, the implementation of techniques unveiling cryptic splice and non-coding variations in RP genes, along with copy number variation (CNV) assays and NGS (WGS with RNA-seq or WES plus Sanger), will establish a genetic diagnosis in >90% of DBA cases [21]. Splice site variants represent ~6% of identifiable genetic defects in DBA. At the same time, their prevalence is doubled when focusing on P/LP variants (14.3%), thus highlighting the impact of such alterations in RP translation and, eventually, in ribosome levels. This study reports the first pathogenic splice site variant in *RPS17* and the eighth in *RPS26*. In silico analysis of the splicing defect was conducted using Genomnis software (https://hsf.genomnis.com/, accessed on 17 October 2023; Human Splicing Finder Professional), the Human Splicing Finder Matrix, and the MaxEnt algorithm. In both variants, the results indicated a broken wild-type acceptor site and an altered wild-type donor site as well as the activation of a cryptic donor site, most probably affecting splicing (Supplementary Figure S1).

The determinants of spontaneous remission and cancer development, the variable expression of the same variants between families, and the selectivity of RP defects towards the erythroid lineage remain to be elucidated with the help of growing DBA consortia and networks.

## Figures and Tables

**Table 1:** The genetic landscape of DBA in RP genes.

Gene	Phenotype	New RP Nomenclature (Chain within 6IP5 Assembly)	No. of Variations Associated with DBA (% of RP Variations; % of P/LP Variants) [9]	Phenotypic Associations (Other than DBA)
***RPS19***	DBA1	eS19 (20)	178 (13.2%; **25%**)	Short stature, failure to thrive, head/neck abnormalities (microcephaly, delayed fontanel closure, micro/retrognathia, hypertelorism, strabismus, flat nose, cleft lip/palate), cardiovascular defects (atrial-septal defect, coarctation of the aorta, absent radial pulse), chest deformities (missing pair of ribs, clavicle agenesis), bone hypoplasia
***RPL5***	DBA6	uL18 (1G)	212 (15.8%; **25%**)	Growth retardation, failure to thrive, hand malformations, cleft lip or palate, genitourinary malformations, cardiovascular malformations
***RPL11***	DBA7	uL5 (2E)	124 (9.2%; **15%**)	Growth retardation, genitourinary malformations, Cathie facies, auditory canal atresia, cleft palate, heart defects, Sprengel deformity, osteopenia, bone deformities
***RPS26***	DBA10	eS26 (3C)	98 (7.3%; **13%**)	Variable expressivity, even within families: growth retardation, facial deformities (mandibular dysostosis, micrognathia, malar hypoplasia, microtia, external auditory canal atresia, low-set ears, choanal atresia, cleft palate), cardiovascular defects, diaphragmatic hernia, genitourinary malformations (duplicated kidney, renal ectopia)
***RPL35A***	DBA5	eL33 (2Z)	84 (6.2%; **5%**)	Short stature
***RPS24***	DBA3	eS24 (3N)	105 (7.8%; **5%**)	Webbed neck
*RPS10*	DBA9	eS10 (2u)	101 (7.5%; 3.1%)	Growth retardation, Cathie facies, webbed neck
*RPS17*	DBA4	eS17 (2y)	15 (1.1%; 2.8%)	Short stature, growth retardation, facial dysmorphism, atrial septal defect, flat thenar
*RPS7*	DBA8	eS7 (2s)	121 (9%; 2.1%)	Short stature, growth retardation, Cathie facies, short nose–broad nasal bridge
*RPL15*	DBA12	eL15 (2H)	10 (0.7%; 2.1%)	Ventricular septal defect, ambiguous genitalia, duplex kidney, triphalangeal thumbs, impaired intellectual development and developmental delay, cerebellar hypoplasia
*RPL26*	DBA11	uL24 (2S)	55 (4.1%; 0.9%)	Short stature, narrowed or absent external auditory meatus, incomplete lower eyelid, cleft palate, bicuspid aortic valve, unilateral hypoplasia/aplasia of the radius, unilateral radioulnar synostosis, hand deformities (absent thumb, bilateral missing digits)
*RPS29*	DBA13	uS14 (3E)	5 (0.4%; 0.9%)	
*RPS28*	DBA15 with MFD	eS28 (3D)	2 (0.1%; 0.9%)	
*RPS20*	DBA uncategorized	uS10 (21)	2 (0.1%; 0.6%)	
*RPL27*	DBA16	eL27 (2T)	4 (0.3%; 0.3%)	
*RPS27*	DBA17	eS27 (3P)	2 (0.1%; 0.3%)	Abnormal skin pigmentation
*RPL35*	DBA19	uL29 (2b)	1 (0.1%; 0.3%)	
*RPL18*	DBA18	eL18 (2K)	1 (0.1%; 0.3%)	
*RPS15A*	DBA20	uS8 (3M)	1 (0.1%; 0.3%)	
*RPL19*	DBA uncategorized	eL19 (2L)	11 (0.8%; none)	
*RPL9* ^^^	DBA1	uL6	1	
*RPLP0* ^^^^	DBA uncategorized	uL10	1	

^^^ not in ClinVar—retrieved from Lezzerini et al., 2020 [10]; ^^^^ not in ClinVar—retrieved from Ulirsch et al., 2018 [3]; MFD: mandibulofacial dysostosis; DBA2 is suggested to reside in the 26.4-cM telomeric region of human chromosome 8p23.3-p22, most likely within an 8.1 cM interval flanked by D8S518 and D8S1825 [11].

**Table 2:** The genetic landscape of DBA-like disorders.

Gene	DBA-Associated P/LP Variations/ Total Variations in Gene	Variation Types	Phenotypic Associations (Other than DBA)
*HEATR3* ^†^	5/5 (100%)	SNVsc.1337G>A (p.Cys446Tyr), c.1751G>A (p.Gly584Glu), c.400T>C (p.Cys134Arg), c.719C>T (p.Pro240Leu), and c.399+1G>T	BMF; short stature, facial and acromelic dysmorphic features; intellectual disability
*TSR2* ^‡^	1/1 (100%)	SNVc.234T>C (p.Asp78=)	MFD
*GATA1*	26/50 (52%)	62% frameshift, 15% missense, 11.5% nonsense, and 11.5% splice site variants	X-linked thrombocytopenia with or without dyserythropoietic anemia; transient myeloproliferative syndrome; acute megakaryoblastic leukemia in Down syndrome
*EPO*	1/3 (33.3%)	SNVc.530G>A (p.Arg177Gln)	Familiar erythrocytosis 5
*ZNF699*	1/6 (16.7%)	SNVc.175+1G>A	DEGCAGS syndrome
*IKZF1*	1/14 (7.1%)	SNVc.1267C>T (p.Arg423Cys)	CVID13; ALL
*ADA2* (alias *CECR1*)	3/54 (5.6%)	SNVc.336C>G (p.His112Gln), 7 bp deletion c.1397_1403del, and 1 bp deletion c.1082-1113del	VAIHS; Sneddon syndrome
*MYSM1*	1/19 (5.3%)	SNVc.1432C>T (p.Arg478Ter)	BMF syndrome 4
*SLC25A38* ^^^	2/50 (4%)	Recessive chr3:39431108:G>T splice donor and chr3:39436065:A>T stop gained	Sideroblastic anemia
*NHEJ1* ^^^	1/26 (3.8%)	Recessive chr2:220011458:G>A stop gained	Cernunnos-XLF deficiency
*PUS1* ^^^	1/27 (3.7%)	Compoundheterozygouschr12:132414269:T>G and chr12:132426447:C>CT (stop gained, frameshift)	Myopathy, lactic acidosis, and sideroblastic anemia
*MPL* ^¶^	1/141 (0.7%)	SNVc.1666G>T (p.Val556Phe)	Myelofibrosis with myeloid metaplasia; congenital amegakaryocytic thrombocytopenia; thrombocythemia; MPD
*TP53*	2/773 (0.3%)	1 bp deletions c.1077del (p.Ser362fs) and c.1083del (p.Ser362fs)	BMF syndrome 5; dyskeratosis congenita
*BMPR2*, *NOP58*	1/457 (0.2%)	Copy number gain GRCh37/hg19 2q33.1-33.2(chr2:203134839-203358214)x3	Pulmonary arterial hypertension; pulmonary veno-occlusive disease
*DAAM1*	1 benign	239.2 kb copy number lossGRCh37/hg19 14q23.1(chr14:59636409-59875592)x1	Heart defects

^^^ not in ClinVar—retrieved from Ulirsch et al., 2018 [3]; ^†^ DBA21 [12]; ^‡^ DBA14 with MFD [13]; ^¶^ retrieved from Pospisilova et al., 2004 [14]; ALL: acute lymphoblastic leukemia; CVID13: common variable immunodeficiency-13; DEGCAGS: developmental delay with gastrointestinal, cardiovascular, genitourinary, and skeletal abnormalities; MPD: myeloproliferative disorder; SNV: single-nucleotide variant; VAIHS: vasculitis, autoinflammation, immunodeficiency, and hematologic defects syndrome.

## Data Availability

The data used to support the findings of this study are available from the corresponding author upon request.

## Supplementary Materials

The following supporting information can be downloaded at: https://www.mdpi.com/article/10.3390/children10111812/s1, Figure S1: (A) Mutation analysis for *RPS17* variant; (B) mutation analysis for *RPS26* variant.
