# Supplementary material for: The Diverse Genomic Landscape of Diamond–Blackfan Anemia: Two Novel Variants and a Mini-Review
Source: Children (Basel). 2023 Nov 15;10(11):1812. doi: 10.3390/children10111812 (PMC10670567; doi:10.3390/children10111812)

Supplementary Figure S1.

(A) Mutation analysis for *RPS17* variant

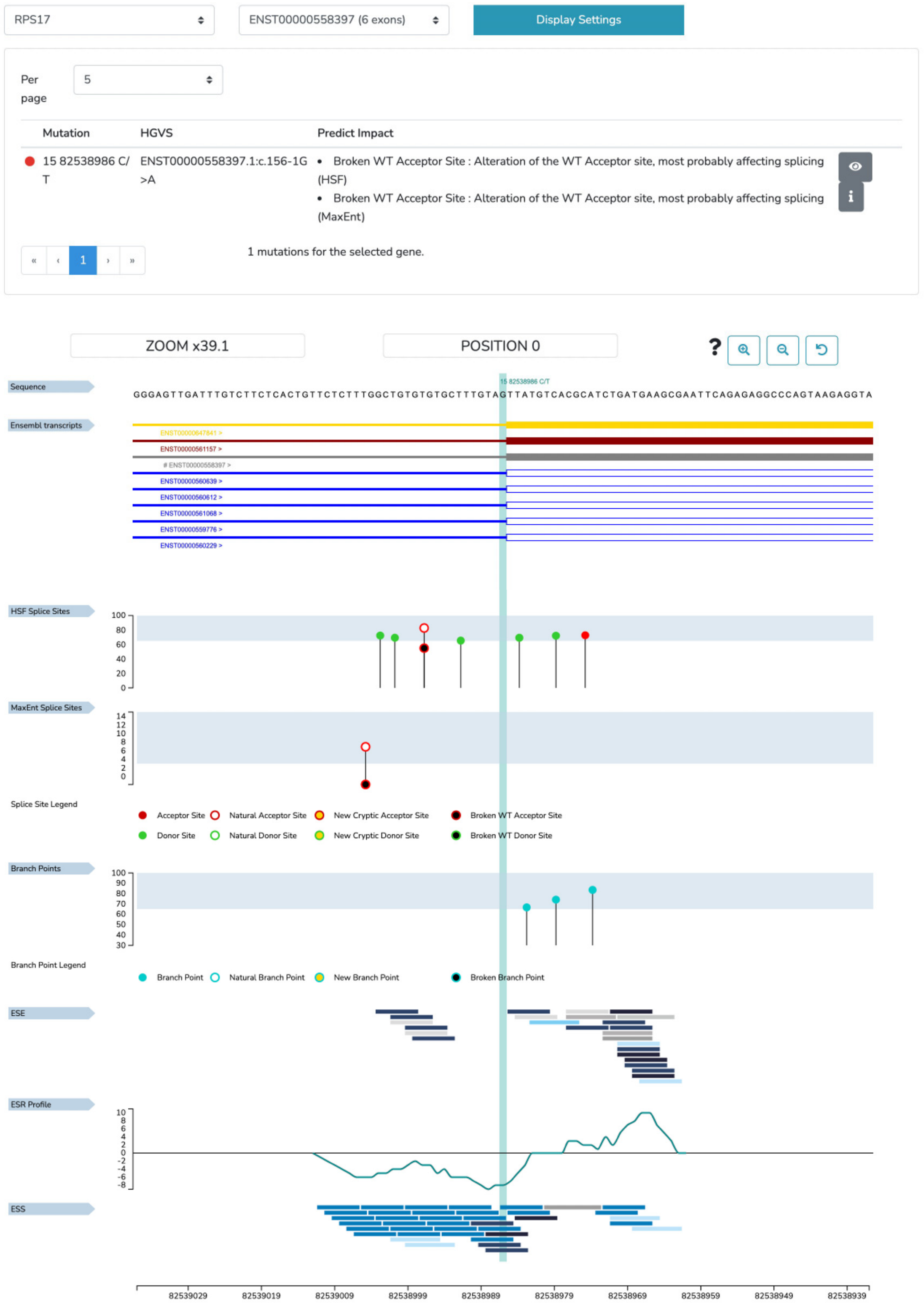

(B) Mutation analysis for *RPS26* variant

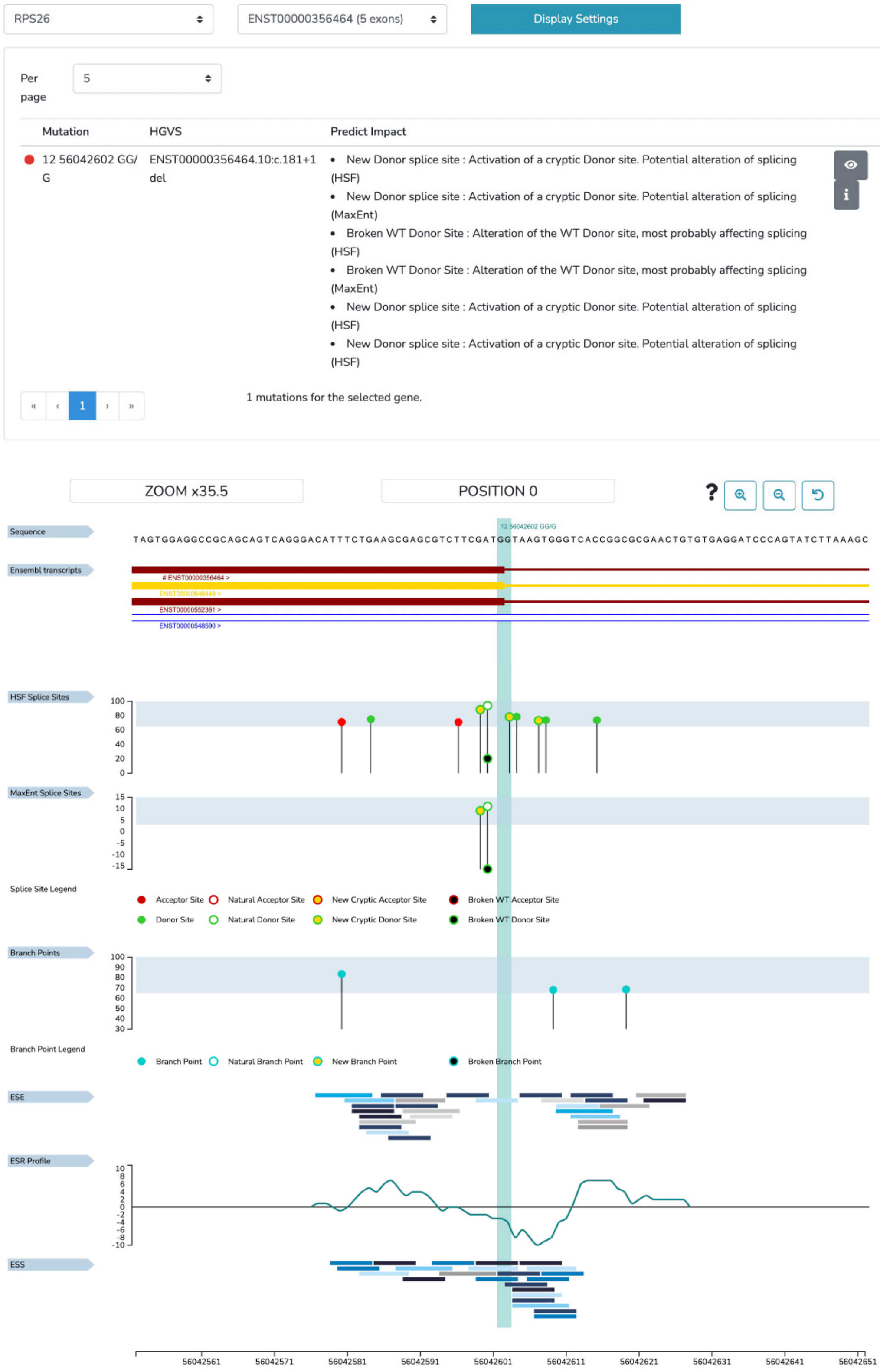

Supplement: Supplementary file 1 [file children-10-01812-s001.zip › children-2705264-supplementary.pdf]
